# Overexpression of Macrophage Migration Inhibitory Factor and Its Homologue D-Dopachrome Tautomerase as Negative Prognostic Factor in Neuroblastoma

**DOI:** 10.3390/brainsci9100284

**Published:** 2019-10-19

**Authors:** Eugenio Cavalli, Emanuela Mazzon, Santa Mammana, Maria Sofia Basile, Salvo Danilo Lombardo, Katia Mangano, Placido Bramanti, Ferdinando Nicoletti, Paolo Fagone, Maria Cristina Petralia

**Affiliations:** 1 IRCCS Centro Neurolesi Bonino Pulejo, C.da Casazza, 98124 Messina, Italy; eugenio.cavalli@irccsme.it (E.C.); emanuela.mazzon@irccsme.it (E.M.); santa.mammana@irccsme.it (S.M.); placido.bramanti@irccsme.it (P.B.); m.cristinapetralia@gmail.com (M.C.P.); 2 Department of Biomedical and Biotechnological Sciences, University of Catania, 95123 Catania, Italy; sofiabasile@hotmail.it (M.S.B.); salvo.lombardo.sdl@gmail.com (S.D.L.); kmangano@unict.it (K.M.); paolofagone@yahoo.it (P.F.)

**Keywords:** Neuroblastoma, MIF, DDT

## Abstract

Neuroblastoma (NB) represents one of the most frequent pediatric solid tumors. Macrophage migration inhibitory factor (MIF) is a cytokine exerting multiple biological functions. More recently, a second member of the MIF family of cytokine has been identified, the D-dopachrome tautomerase (DDT), that exerts several overlapping functions with MIF. Growing evidence suggests a key role for MIF and DDT in the development of cancer. The aim of this study is to characterize the prognostic value of MIF and DDT in NB. We show that higher expression levels of MIF and DDT in Stage 4 NB samples are associated with a poorer prognosis, independently of the presence of MYCN amplification. Moreover, higher levels of MIF are mostly enriched by Th1 cells, while lower levels of MIF are associated with an increased proportion of B cells, Cytotoxic T cells, Dendritic cells and Natural Killer T cells. We also show that treatment with the histone deacetylase (HDAC) inhibitor, vorinostat, of the NB cell line, SH-SY5Y, determines a significant reduction in the expression of both MIF and DDT. Finally, MIF and DDT inhibition by short interfering RNA is able to revert vincristine sensitivity in vitro. Overall, our data suggest that MIF exert pro-tumorigenic properties in NB, likely by dampening antigen presentation and cytotoxic immune responses, and we propose the HDAC inhibitors as a potential therapeutic strategy for NB patients.

## 1. Introduction

Neuroblastoma (NB) is a neoplasm originating from neural crest cells and represents one of the most frequent pediatric solid tumors, as it accounts for 7% of malignancies diagnosed in children from 0 to 14 years of age. NB is responsible for nearly 15% of pediatric cancer-related mortality [1]. The International Neuroblastoma Staging Series (INSS) classifies NB patients by risk level, tumor location and dissemination, and MYCN amplification [2]. The standard of care (SOC) for NB consists of surgery, chemo-radiotherapy, and more recently, antidisialoganglioside (anti-GD2) immunotherapy [3,4]. While the 5-year survival rate for patients with low-risk NB is higher than 95%, it drops to 40%–50% in high-risk patients [5]. Results from a recent Phase 3 trial (NCT00567567) have shown that high-risk neuroblastoma patients treated with tandem autologous stem-cell transplant with thiotepa/cyclophosphamide followed by dose-reduced carboplatin/etoposide/melphalan had significantly better event-free survival (EFS) when compared to patients treated with a single transplant with carboplatin/etoposide/melphalan [6]. However, despite recent advancement in the therapeutic strategies for high-risk neuroblastoma patients, more target-specific approaches are warranted for this latter group of patients. 

Macrophage migration inhibitory factor (MIF) is a pro-inflammatory cytokine expressed by several cellular types, including epithelial, endothelial and immune cells [7]. MIF binds the Human Leukocyte Antigen (HLA) class II histocompatibility antigen gamma chain, CD74, causing its phosphorylation and the recruitment of CD44, which allows the activation of the Extracellular-Signal-Regulated Kinase (ERK)- Mitogen Activated Protein Kinase (MAPK) pathway. Also, MIF is a non-cognate ligand for the chemokine receptors, C-X-C chemokine receptor type (CXCR) 2, 4, 7, and binds the intracellular receptor C-Jun activation domain-binding protein-1 (JAB1) [8]. A second member of the MIF superfamily has been characterized, the D-dopachrome tautomerase (DDT, also known as MIF-2) [9]. Similar to MIF, DDT binds CD74, although with a 3-fold higher acid dissociation constant and a 11-fold higher dissociation rate. On the other hand, DDT lacks the motif that allows MIF binding to the chemokine receptor, CXCR2 [7].

In addition of being implicated in the pathogenesis of autoimmune diseases [7], such as Multiple Sclerosis [10,11,12], Guillain Barrè Syndrome [13] and Type 1 Diabetes [14], MIF has been shown to promote tumorigenesis [15] and it has been described to be overexpressed by various tumors, including mammary [16], colorectal and prostate cancer [17], melanoma [18] and glioblastoma [19,20]. Indeed, MIF may induce angiogenesis by up-regulating the secretion of vascular endothelial growth factor (VEGF) [18], may promote proliferation, by binding and deactivating JAB1, a negative regulator of p27KIP1, that in turn controls cell cycle progression at the G1 phase [21]; and may inhibit natural killer cells-mediated cytotoxicity [22]. Finally, MIF and DDT have been shown to synergistically inhibit steady state p53 phosphorylation stabilization and transcriptional activity [23].

In the present study, we aimed at evaluating the prognostic role of MIF in NB and exploring potential therapeutic strategies for its modulation. 

## 2. Materials and Methods

### 2.1. Dataset Selection and Analysis

RNA Seq data were obtained from The Cancer Genome Atlas (TCGA) datasets through the cBioportal web-based utility [24]. The dataset comprised 111 primary INSS Stage 4 tumors and clinical data included overall survival time, presence of MYCN amplification, Mitosis–Karyorrhexis index (MKI) and histology grade. Patients were stratified in quartiles based on the expression of MIF and DDT genes and samples in the upper and lower quartiles were selected for comparison.

### 2.2. Computational Deconvolution of Infiltrating Immune Cells

In order to evaluate the relative proportions of the infiltrating immune cell subsets in NB samples diverging for the expression of MIF and stratified in accordance to Section 2.1, we performed a computational deconvolution analysis. To this aim, we have used the web-based utility, xCell [25], a computational tool that is able, by using gene signatures, to infer the presence in a sample of various cell types, including active Dendritic Cells (aDCs), astrocytes, B cells, CD4+ naive T cells, conventional DCs (cDCs), memory B cells, plasma cells, Th1 cells, Th2 and Treg cells and monocytes/macrophages [26].

### 2.3. Effect of Vorinostat Treatment on Migration Inhibitory Factor (MIF) Expression 

It was previously shown that histone deacetylase inhibitors strongly inhibit MIF expression in a variety of cell lines, primary cells and in vivo [27]. For the evaluation of the impact of the histone deacetylase inhibitor, vorinostat, on MIF and DDT expression, we interrogated the GSE49158 dataset [28]. To generate the dataset, SH-SY5Y cells were treated with either Dimethyl sulfoxide (DMSO) or 10 µM of vorinostat for 6 h and 24 h, or pulsed with vorinostat for 6 h followed by cellular wash and drug-free media replacement, for further 18 h later (24 h from initial compound treatment). Total RNA was extracted and hybridized onto Agilent 4 × 44,000 Whole Human Genome microarrays. Background correction of raw data was performed using the normexp function in the limma package and within-array normalization was performed using the normalizeWithinArrays function with the loess method. Differential analysis of expression for the genes of interest was performed using LIMMA software and Benjamini–Hochberg’s adjusted *p*-values < 0.05 and a ǀfold change׀ > 2 was considered as threshold for statistical significance.

### 2.4. Effect of MIF and D-Dopachrome Tautomerase (DDT) on Vincristine Resistance In Vitro

The neuroblastoma cell line UKF-NB-3 and the UKF-NB-3 sub-line adapted to 10 ng/mL vincristine (VCR10) were established as previously described [29]. VCR10 cells were transfected with double-stranded siRNAs (dsRNA) targeting the MIF gene (NM_002415) or the DDT gene (NM_001084392), or with corresponding scrambled siRNAs, using Lipofectamine 2000 reagent in OPTI-MEM serum-free medium, with a final siRNA concentration of 100 nM. The cells were collected after 48 h of siRNA transfection and exposed to 5 scalar ten-fold concentrations of vincristine (range: 0.01–100 ng/mL). The parental cell line UKF-NB-3 was used as control. Cell viability was tested by the 3-(4,5-dimethylthiazol-2-yl)-2,5-diphenyltetrazoliumbromide (MTT) dye reduction assay after 48 h of incubation. All experiments were performed in triplicate.

### 2.5. Statistical Analysis

Gene expression differences were evaluated using one-way analysis of variance (ANOVA), followed by Bonferrori post hoc test. Correlation analysis was performed using both the parametric Pearson’s test and the non-parametric Spearman’s test. Survival analysis was performed using Kaplan–Meier and its significance analyzed by the log-rank (Mantel–Cox) test. Predicted values were obtained by a general linear model using MYCN amplification as fixed factor. For the analysis, a *p*-value < 0.05 was considered statistically significant. Statistical analysis was performed with GraphPad Prism 8 (GraphPad Software, San Diego, CA, USA) and SPSS 24 (IBM SPSS Statistics, IBM Corporation, Armonk, NY, USA).

## 3. Results

### 3.1. Expression of MIF in Neuroblastoma (NB)

**Figure 1:**
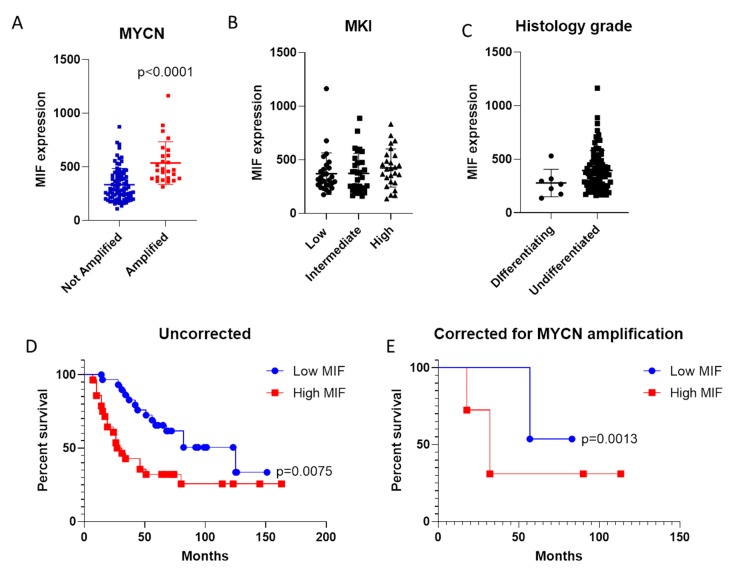
Expression of macrophage migration inhibitory factor (MIF) in neuroblastoma (NB). **A**. Evaluation of MIF transcriptional levels in Stage 4 NB samples with and without MYCN amplification; **B**. MIF expression levels in Stage 4 NB samples with different Mitosis–Karyorrhexis index (MKI) score; **C**. MIF levels in Stage 4 NB samples at different histological grades; **D**. Overall Survival curve for Stage 4 NB patients with high and low expression levels of MIF; **E**. Overall Survival curve for Stage 4 NB patients with high and low expression levels of MIF, accounting for the presence of MYCN amplification.

The expression levels of MIF in Stage 4 NB samples were determined by interrogating the TCGA dataset. As shown in Figure 1, MIF expression levels resulted significantly higher in samples bearing the MYCN amplification (*p* < 0.0001; Figure 1A). No significant differences were instead observed among samples with various MKI score (Figure 1B). The histology grade was not associated to a significant modulation in MIF expression levels, although a trend of higher MIF levels was instead observed in samples characterized by poorly differentiated/undifferentiated cells (Figure 1C). 

### 3.2. Evaluation of MIF as Negative Prognostic Factor in NB

Patients were stratified based on the transcriptomic levels of MIF, and a survival curve was constructed for overall survival (OS). As shown in Figure 1D, a better OS was observed for patients expressing lower levels of MIF (*p* = 0.0075; Figure 1D). Interestingly, lower levels of MIF were associated to better OS, even after correcting for the presence of MYCN amplification (*p* = 0.0013; Figure 1E).

### 3.3. Prognostic Value of DDT in NB

**Figure 2:**
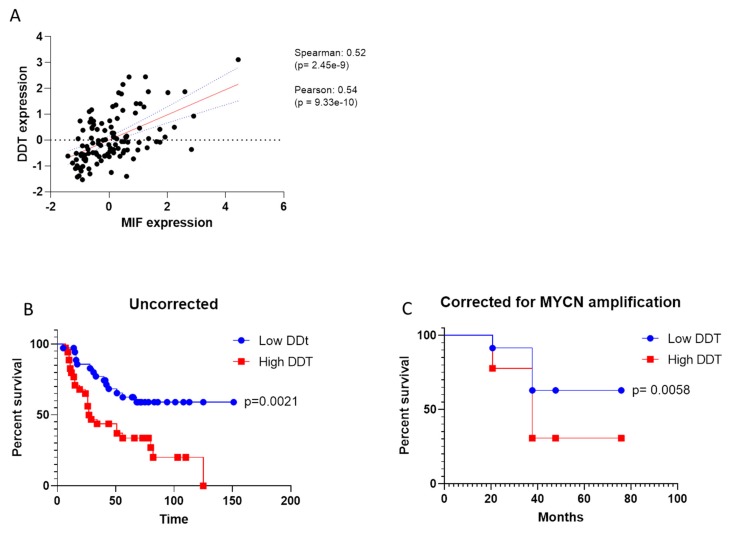
Evaluation of D-dopachrome tautomerase (DDT) in neuroblastoma (NB). **A**. Correlation between MIF and DDT levels in Stage 4 NB samples (gene expression is defined as arbitrary unit); **B**. Overall survival curve for Stage 4 NB patients with high and low expression levels of DDT; **C**. Overall survival curve for Stage 4 NB patients with high and low expression levels of DDT, accounting for the presence of MYCN amplification.

As the second member of MIF superfamily, the correlation between DDT and MIF was evaluated in Stage 4 NB samples, by using the Spearman’s test. A strong positive correlation was observed, which entailed a *p* = 2.45× e^−9^ (Figure 2A). A log-rank test was performed to evaluate the impact of DDT expression on OS and, according to the data on MIF, lower levels of DDT accounted for better OS, irrespective of the presence of MYCN amplification (Figure 2A,B).

### 3.4. Deconvolution Analysis

**Figure 3:**
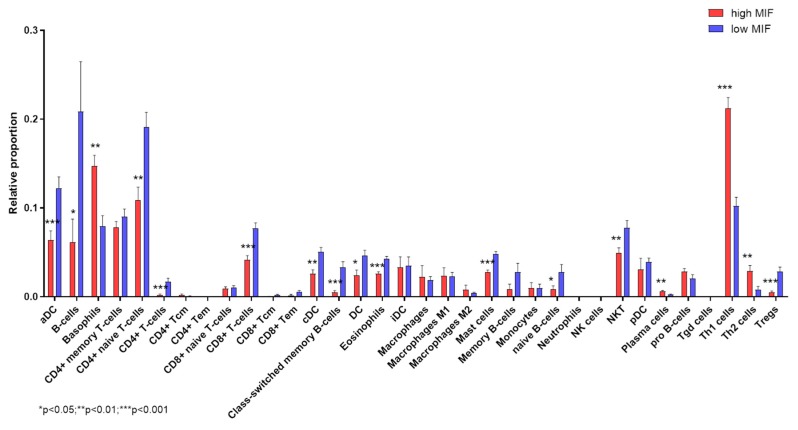
Deconvolution analysis of infiltrating immune cells in neuroblastoma (NB). Infiltrating immune cell populations were predicted using the web-based deconvolution analysis utility, xCell, for Stage 4 NB patients with high and low expression levels of MIF.

Deconvolution analysis of cell infiltration in Stage 4 NB samples, dichotomized on MIF expression levels, revealed that higher MIF levels are associated with significant lower proportions of infiltrating CD4 T naïve cells. Along with this, a significantly higher proportion of Th1 and Th2 cells, and lower Treg cell infiltration was observed (Figure 3). On the other hand, samples with low expression levels of MIF were characterized by a significant lower infiltration of DCs (both aDCs and cDCs), B cells (including class-switched memory B cells), CD8+ T cells and Natural Killer T (NKT) cells (Figure 3). A significant higher infiltration of basophils, along with reduced proportions of mast cells and eosinophils, were observed in NB samples with high MIF expression (Figure 3). 

### 3.5. Vorinostat Effect on MIF Expression in NB

**Figure 4:**
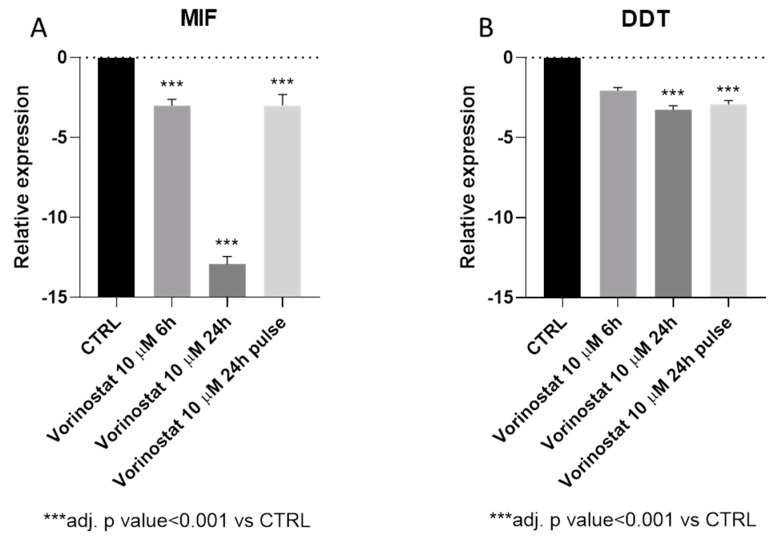
Effect of vorinostat on macrophage migration inhibitory factor (MIF) and D-dopachrome tautomerase (DDT) expression. The modulation of MIF (**A**) and DDT (**B**) upon treatment with vorinostat of the neuroblastoma cell line, SH-SY5Y, by interrogating the publicly-available, GSE49158 microarray dataset.

The impact of vorinostat on MIF and DDT expression was evaluated by analyzing the GSE49158 microarray dataset. As shown in Figure 4A, treatment of the NB cell line SH-SY5Y with 10 µM vorinostat determined a significant reduction in MIF expression levels, already at 6h post-treatment. At 24 h post-treatment, a 12.9-fold reduction in MIF levels could also be observed (Figure 4A). Interestingly, the effect of vorinostat on MIF expression was maintained after 18 h of incubation in drug-free medium, following 6 h of treatment (pulsed treatment) (Figure 4A). Along the same lines, DDT transcriptional levels were reduced upon vorinostat treatment, although the statistical significance was only reached at the 24h time-point (3.3-fold reduction) (Figure 4B).

### 3.6. Effect of MIF and DDT on Vincristine Resistance In Vitro

**Figure 5:**
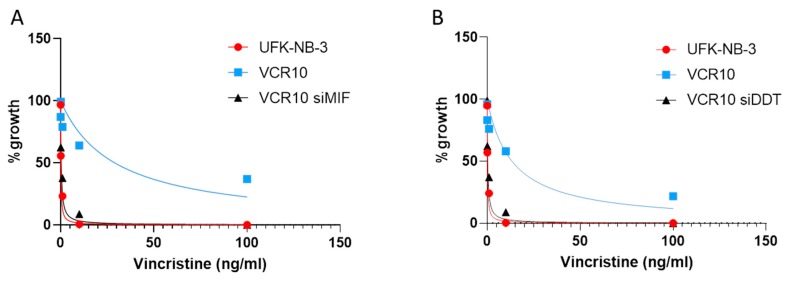
Effect of macrophage migration inhibitory factor (MIF) and D-dopachrome tautomerase (DDT) inhibition on vincristine resistance. The human neuroblastoma cell line UKF-NB-3 adapted to 10 ng/mL vincristine (VCR10) was transfected with double-stranded siRNAs (dsRNA) targeting the MIF gene (*NM_002415*) (**A**) or the DDT gene (*NM_001084392*) (**B**), and corresponding scrambled siRNAs, and exposed to 5 scalar ten-fold concentrations of vincristine (range: 0.01–100 ng/mL). Cell viability was tested by the 3-(4,5-dimethylthiazol-2-yl)-2,5-diphenyltetrazoliumbromide (MTT) dye reduction assay after 48 h of incubation. The parental UFK-NB-3 cell line was used as control. All experiments were performed in triplicate.

The influence of MIF and DDT on vincristine sensitivity was evaluated in the vincristine-resistant neuroblastoma cells line VCR10. As shown in Figure 5A, MIF inhibition was associated with a significant reduction in vincristine IC50 (*p* < 0.001 vs. scrambled siRNA control cells). Along the same lines, DDT siRNA restored vincristine sensitivity (*p* < 0.05 vs. scrambled siRNA control cells) (Figure 5B).

## 4. Discussion

The property of MIF and DDT that enables them to control fundamental cellular processes, such as proliferation and invasion of tumour cells [17], along with the possibility of specific pharmacological inhibition of MIF by small molecules or monoclonal antibody directed against the cytokine or its receptor [7,30,31], has recently focused much attention on the possible use of MIF and DDT as theranostic molecules that may be useful both for diagnostic purposes and as novel chemotherapeutic targets. Although less data are available for DDT, which has been discovered more recently, several preclinical and clinical data have concordantly shown high levels of MIF in a variety of human cancers [32,33,34], including pancreatic and gastric cancer, melanoma, hepatocarcinoma, glioma and cervical adenocarcinoma [30]. Moreover, tumor expression and/or circulating plasma levels of MIF have been proposed as biomarkers of prognosis and therapeutic response [35,36,37,38,39,40,41]. 

Ren and colleagues have shown that inhibition of MIF expression, via antisense cDNA, in NB cells determined a significant reduction in tumor growth in vitro and tumor metastasis in vivo [42].

In the present study, we aimed at characterizing the prognostic value of MIF in NB and at identifying potential therapeutic approaches able to modulate its expression. This analysis has been carried out by using publicly available whole-genome transcriptomic databases that represent useful in silico tools for the better understanding of pathogenic pathways and the possible prediction of novel diagnostic therapeutic approaches in a broad range of clinical settings, including immunoinflammatory/autoimmune disorders [11,12,43,44,45,46], cancer [47,48,49,50,51,52,53], fibrotic [54,55], neurological and neuropsychiatric diseases [56,57] and identification of cellular and molecular therapeutic targets [58].

Genomic amplification of MYCN is found in approximately 20% of all NB cases and it is strictly associated with poor prognosis [59,60]. MYCN belongs to the MYC family of transcription factors, which are key regulators of a broad range of cellular processes, i.e. survival, proliferation, and differentiation, that may be disrupted in malignant transformation [61,62]. Physiologically, a high MYCN expression is observed during embryogenesis and its expression is generally low in adult tissues [63]. In high-risk NB samples, that do not have MYCN amplification, the expression of MYCN is also often increased [64], suggesting a regulatory role for the MYC family in the etiopathogenesis of NB tumorigenesis. The relevance of MYCN in NB has also attracted interest in identifying specific inhibitors of this gene as novel therapeutic approaches for the treatment of NB. Along this line of research, Wang et al. have shown that the combination of two Food and Drug Administration (FDA)-approved drugs entailing the proteasome inhibitor bortezomib (BTZ) and the histone deacetylase (HDAC) inhibitor vorinostat concertedly induce dramatic cell death in NB cell lines in part through synergistic activation of BAX in MYCN transformed NB cell lines [65]. As vorinostat, that is approved for the treatment of Cutaneous T Cell Lymphoma (CTCL) since 2006, has been shown to inhibit MIF production we wondered whether the in vitro chemotherapeutic action of this drug in MYCN transformed NB cell lines could be associated to down-modulation of MIF and DDT [66].

In the present study, we have observed that NB samples bearing the MYCN amplification have significantly higher levels of MIF and a trend of increase of DDT (data not shown). However, it should be noted that a (Chromatin ImmunoPrecipitation (ChIP)-chip analysis did not show the presence of binding sites for MYCN at the MIF and DDT promoters [67]. Chia-Lang Hsu and collaborators have previously shown that although only approximately 40% of MYCN-correlated genes are bound by MYCN, as many of them are transcription factors, each MYCN-correlated gene resulted co-expressed with at least one of them [68]. Therefore, it is reasonable that MYCN may indirectly regulate MIF expression. Yao and collaborators demonstrated that the transcription factor, ICBP90, is a key regulator of MIF expression in both immune cells and synovial fibroblasts, and that Toll-like receptor (TLR)-induced MIF transcription is controlled in an ICBP90- and -794 CATT5-8 length-dependent manner [69]. Therefore, more in depth studies on the regulation of MIF transcription needs to be performed, for the possible development of targeted therapies for the treatment of NB patients. 

It is also worth mentioning that, from the survival analysis, MIF resulted in being an independent negative prognostic factor for stage 4 NB patients. In accordance with the strong and significant correlation in the expression levels, also the second member of the MIF superfamily, DDT has been found to negatively influence the OS, independently of MYCN amplification. DDT and MIF share a similar tertiary structure and both exert tautomerase activity [9]. Our data seem to suggest that MIF and DDT may have overlapping effects on NB tumorigenesis. Along the same lines, in non-small cell lung carcinoma, MIF and DDT have been shown to cooperate in regulating angiogenesis, by inducing CXCL8 and VEGF production, and a functional redundancy for MIF and DDT has been also shown in promoting tumor growth and cell migration, in clear renal cell carcinomas [70,71].

We also show here that the histone deacetylase inhibitor (HDI), vorinostat, is able in vitro to significantly regulate the expression of MIF and DDT in a human NB cell line. Our data follows a report by Lugrin and coworkers [27] that demonstrated that trichostatin A (TSA) reduced MIF transcription in HeLa epithelial cells in a time- and dose-dependent manner, similarly to other HDIs, including vorinostat and valproic acid (VPA). Moreover, TSA significantly inhibited MIF expression also in HaCat keratinocytes, HL-60, KG1a and U-937 leukemic cell lines, THP-1 monocytic cells, A549 airway epithelial cells and B16 melanoma. Also, when administered to mice TSA mice reduced by two-fold the circulating levels of MIF. Interestingly, by using nuclear run-on assays TSA was shown to directly reduce MIF mRNA expression by inhibiting its transcription, and by affecting the level of acetylated histones H3 and H4 specifically associated with the MIF promoter [27]. However, despite the difficulty in discerning the causality link between MIF modulation and neuroblastoma phenotype upon vorinostat, it should be pointed out that identifying drugs, already approved for clinical use, that are able to modulate MIF and DDT, is particularly important as MIF-targeted therapies are still in preclinical or in very early clinical development. Also, no DDT-targeted drugs are yet available. Hence, we may speculate that patients suffering from disorders characterized by a dysfunction of the MIF/DDT pathway may benefit from the use of drugs, such as vorinostat, able to dampen the expression of these genes.

We have also shown that the inhibition of the expression of both MIF and DDT, by short interfering RNA, in vincristine-resistant NKF-NB-3 neuroblastoma cells, is able to revert drug resistance. Although the characterization of the molecular mechanisms behind this observation are beside the scope of the present paper, we may speculate that MIF/DDT inhibition could improve the activity of p53, inhibiting cell-cycle progression, anchorage independence, and increasing programmed cell death, as previously shown in human lung adenocarcinoma cell lines by [23] and, consequently reducing the IC50 of vincristine.

Recent data suggest that MIF may promote tumorigenesis by favoring the escape of malignant cells from immune surveillance, via the induction of myeloid-derived suppressor cells [72], the inhibition of T lymphocyte activation [73], and polarization of macrophages to an M1 phenotype [74]. Finally, MIF has been shown to inhibit natural killer (NK) cell-mediated cytotoxicity [22]. Our deconvolution analysis of immune cell infiltration revealed that samples with higher levels of MIF are mostly enriched by Th1 cells, while lower levels of MIF are associated to an increased proportion of B cells, CTLs, DCs and NKT cells. We may propose that MIF exert pro-tumorigenic properties in NB, likely by dampening antigen presentation and cytotoxic immune responses.

During the last few years, several small molecule inhibitors of MIF have been shown to exert chemotherapeutic effects in rodent models of cancer. In a similar manner an anti-MIF mAb (BAX69) has been tested in Phase II studies (NCT02540356 and NCT02448810) in cancer patients. The demonstrated evidence of the synergisms of MIF and DDT in the OS of NB study suggest that dual inhibitors of MIF and DDT could have even better therapeutic potential against NB and eventually other types of cancers than a single antagonist. In this regard, the anti-CD74 antibody could be considered that block the interaction of both cytokines with this receptor. The anti-CD74 mAb milatuzumab has already been tested in Phase II studies (NCT00868478 and NCT00989586) for the treatment of chronic lymphocytic leukemia and in combination with the anti-CD20 mAb veltuzumab in relapsed and refractory B-cell non-Hodgkin’s lymphoma. So new studies in the setting of NB could be rapidly carried out. In addition, the first small-molecule dual inhibitor of MIF and DDT that is still in preclinical development has also been characterized and may be worth being studied for its use in NB [75]. Finally, it has been recently observed that the biological activity of MIF may be impaired by nitrosylation [76]. Therefore, we may envisage the possibibility to use nitric oxide (NO)-hybridized drugs, such as NO-aspirin or NO-hybridized antiretroviral protease inhibitors, such as lopinavir-NO [77,78,79,80], for the treatment of MIF-dependent disorders including, neuroblastoma. 

## 5. Conclusions

Our present data not only reveal novel immunopathogenetic pathways of NB that implicate the MIF superfamily but also suggest that MIF and DDT may represent theranostic cytokines that may predict chemotherapeutic responses and allow design tailored target therapeutic approaches based on non-specific or specific MIF and DDT inhibition.
